# A Mechanism for the Activation of the Influenza Virus Transcriptase

**DOI:** 10.1016/j.molcel.2018.05.011

**Published:** 2018-06-21

**Authors:** Itziar Serna Martin, Narin Hengrung, Max Renner, Jane Sharps, Mónica Martínez-Alonso, Simonas Masiulis, Jonathan M. Grimes, Ervin Fodor

**Affiliations:** 1 Sir William Dunn School of Pathology, University of Oxford, South Parks Road, Oxford OX1 3RE, UK; 2 Division of Structural Biology, Henry Wellcome Building for Genomic Medicine, University of Oxford, Oxford OX3 7BN, UK; 3 Diamond Light Source, Harwell Science and Innovation Campus, Didcot OX11 0DE, UK

**Keywords:** influenza virus, RNA polymerase, transcriptase, cap snatching, transcription, replication, Pol II, CTD

## Abstract

Influenza virus RNA polymerase (FluPol), a heterotrimer composed of PB1, PB2, and PA subunits (P3 in influenza C), performs both transcription and replication of the viral RNA genome. For transcription, FluPol interacts with the C-terminal domain (CTD) of RNA polymerase II (Pol II), which enables FluPol to snatch capped RNA primers from nascent host RNAs. Here, we describe the co-crystal structure of influenza C virus polymerase (FluPol_C_) bound to a Ser5-phosphorylated CTD (pS_5_-CTD) peptide. The position of the CTD-binding site at the interface of PB1, P3, and the flexible PB2 C-terminal domains suggests that CTD binding stabilizes the transcription-competent conformation of FluPol. In agreement, both cap snatching and capped primer-dependent transcription initiation by FluPol_C_ are enhanced in the presence of pS_5_-CTD. Mutations of amino acids in the CTD-binding site reduce viral mRNA synthesis. We propose a model for the activation of the influenza virus transcriptase through its association with pS_5_-CTD of Pol II.

## Highlights

• Influenza C virus RNA polymerase binds the CTD of RNA polymerase II
• Pol II CTD binding allows the viral polymerase to snatch capped RNA primers
• Pol II CTD binding stabilizes the transcriptase conformation of the viral polymerase
• Pol II CTD binding enhances viral transcription

## Graphical Abstract

**Figure:**
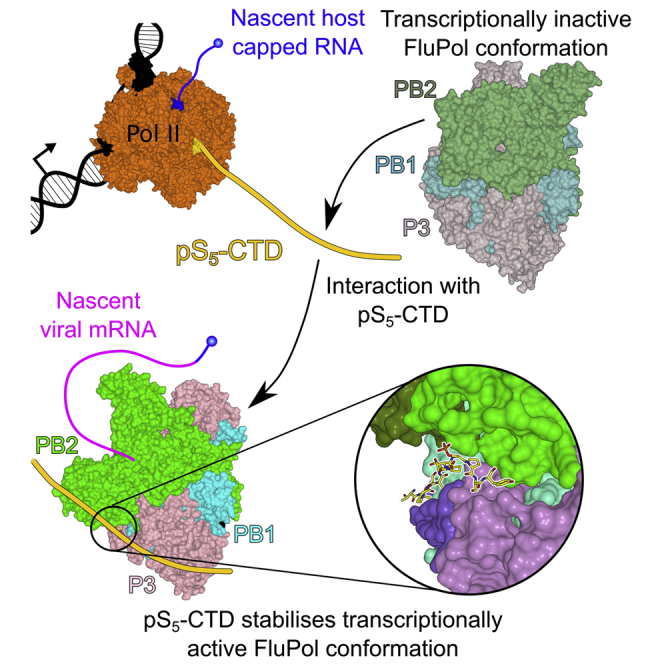

## Teaser

The influenza virus RNA polymerase acts both as transcriptase and replicase. Serna Martin et al. solve the structure of the influenza C virus polymerase bound to a peptide mimicking the C-terminal domain of Pol II and demonstrate that binding to Pol II stabilizes the transcriptase conformation of the viral polymerase.

## Introduction

Influenza viruses contain a segmented single-stranded negative-sense RNA genome, which is assembled into viral ribonucleoprotein (vRNP) complexes. In the vRNP, the 5′ and 3′ terminal sequences of the viral RNA (vRNA) associate with the viral RNA-dependent influenza virus RNA polymerase (FluPol) whereas the rest of the vRNA is bound by oligomeric nucleoprotein (NP) (Arranz et al., 2012, Moeller et al., 2012). FluPol replicates and transcribes the vRNA in the context of vRNPs in the nucleus of the host cell. Replication is a two-step, primer-independent process, requiring *de novo* initiation by FluPol, involving the synthesis of a cRNA replicative intermediate from the vRNA template (step 1) and the synthesis of vRNA from the cRNA template (step 2). On the other hand, transcription is a primer-dependent process that results in capped and polyadenylated mRNAs (Pflug et al., 2017, Te Velthuis and Fodor, 2016). Whereas other negative-sense viruses, such as the *Filoviridae* and *Paramyxoviridae*, synthesize their own cap structures (Decroly et al., 2011), influenza virus obtains these from cellular transcripts, through a process known as cap snatching (Beaton and Krug, 1981, Plotch et al., 1981). During cap snatching, FluPol binds host-capped RNAs through its PB2 cap-binding domain, and its PA endonuclease cleaves these to produce 10- to 14-nt-long capped RNA fragments. These are used by FluPol as primers to initiate viral mRNA synthesis. In order to carry out cap snatching, FluPol needs to assume a transcriptional pre-initiation conformation (Te Velthuis and Fodor, 2016).

Recently obtained high-resolution structures of FluPol reveal that it consists of a central polymerase core domain made up of PB1, the PA C-terminal (PA C-ter) domain and the N-terminal third of PB2 (PB2 N-ter), and several flexible peripheral appendages, including the N-terminal PA endonuclease and the C-terminal two-thirds of PB2 that encompass the cap-binding domain (CBD), mid-link, 627 and nuclear localization signal (NLS) domains (Hengrung et al., 2015, Pflug et al., 2014, Reich et al., 2014). The structure of FluPol has been observed in two major distinct conformations. FluPol_A_ and FluPol_B_ bound to vRNA crystallized in a transcription pre-initiation state with the PB2 cap-binding and PA endonuclease domains aligned for cap snatching (Pflug et al., 2014, Reich et al., 2014). In contrast, the apo form of influenza C virus polymerase (FluPol_C_) and FluPol_B_ bound to the 5′ end of cRNA, crystallized in a transcriptionally inactive conformation, with the PA (P3 in FluPol_C_) endonuclease and C-terminal two thirds of PB2 arranged in a manner incompatible with cap snatching (Hengrung et al., 2015, Thierry et al., 2016). Although interaction with vRNA is required for FluPol to assume the transcription pre-initiation conformation, it remains unclear what additional factors regulate the conformational states of FluPol.

In order to cap snatch, FluPol needs to bind host-capped RNAs, which serve as a source of capped RNA primers for viral mRNA synthesis. However, accessing the caps of nascent cellular transcripts presents a challenge, as these are rapidly sequestered by cellular proteins involved in nuclear export and pre-mRNA processing (Darnell, 2013, Lidschreiber et al., 2013). FluPol is able to gain preferential access to caps by associating with the initiating form of RNA polymerase II (Pol II) (Chan et al., 2006, Engelhardt et al., 2005, Loucaides et al., 2009). This is recognized through a direct and selective interaction with the Ser5-phosphorylated C-terminal domain (CTD) heptad repeat sequence Y_1_S_2_P_3_T_4_pS_5_P_6_S_7_ (Lukarska et al., 2017, Martínez-Alonso et al., 2016), which is the signature for initiating Pol II (Jeronimo et al., 2016). As well as enabling viral transcription, the FluPol-CTD interaction facilitates the suppression of host gene expression by inhibiting Pol II elongation and promoting Pol II degradation (Rodriguez et al., 2007, Vreede et al., 2010).

In FluPol_A_, there are two sites, both within the PA C-terminal domain, that contribute to CTD binding (Lukarska et al., 2017). At site 1, Y_1_ of a CTD repeat is bound in a hydrophobic pocket formed by PA residues F440 and F607, whereas the phosphoserine-5 (pS_5_) is accommodated in a positively charged pocket formed by PA K630 and R633. These residues are conserved in FluPol_B_, and unsurprisingly, FluPol_B_ binds CTD in the same conformation at its equivalent site 1. FluPol_A_ site 2 lies in a groove formed by PA β18, β19, and the connecting 550 loop. However, the binding residues of site 2 are not conserved in FluPol_B_, and in this case, the CTD, which is less well ordered, appears instead to extend across the PB2 627 domain (Lukarska et al., 2017). Neither site 1 nor site 2 residues are conserved in FluPol_C_, raising the question of how FluPol_C_ binds to CTD.

Here, we have crystallized FluPol_C_ bound to a Ser5-phosphorylated Pol II CTD mimic peptide (pS_5_-CTD) consisting of four heptad repeats. We have identified CTD-binding sites, distinct from those observed in FluPol_A_ and FluPol_B_. Furthermore, through a combination of cryoelectron microscopy (cryo-EM) analysis of the vRNA promoter bound FluPol_C_, *in vitro* FluPol_C_ activity assays in the presence of CTD peptides, and cell-based minireplicon assays, we uncover a regulatory mechanism whereby an interaction with the CTD of Pol II is key to the activation of the viral transcriptase.

## Results

### Structure of the pS_5_-CTD-Bound FluPol_C_

**Figure 1:**
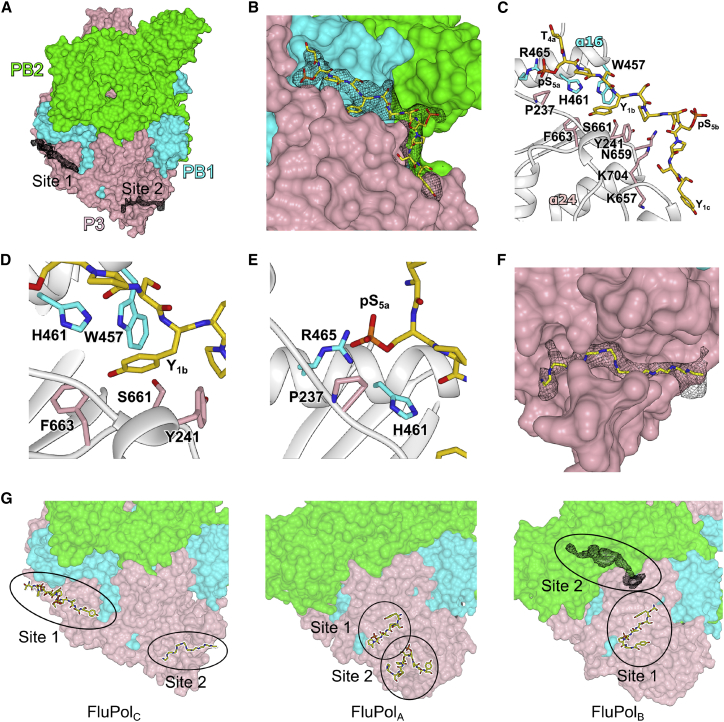
Structure of FluPol_C_ Bound to Pol II CTD Peptide (A) Surface representation of FluPol_C_ crystal structure with electron density maps shown at CTD-binding sites 1 and 2 (site 1: Sigma-A-weighted *2F*_*o*_*-F*_*c*_, 0.9σ, black mesh; site 2: Sigma-A-weighted *F*_*o*_*-F*_*c*_, 2.6σ, black mesh). (B) Binding site 1 close up with the pS_5_-CTD peptide (yellow) modeled in the Sigma-A-weighted *2F*_*o*_*-F*_*c*_ electron density map (0.9σ). (C) Detailed view of the pS_5_-CTD peptide interaction at site 1 with key amino acids highlighted. (D) Detailed view of the hydrophobic pocket that accommodates Y_1b_. (E) Detailed view of the pocket that accommodates pS_5a_. (F) Binding site 2 close up with poly-alanine chain (yellow) backbone shown in the difference electron density map. (G) Comparison of the Pol II CTD-binding sites on FluPol_C_, FluPol_A_ (PDB: 5M3H), and FluPol_B_ (PDB: 5M3J). Site 2 in FluPol_B_ is shown as difference electron density map (Sigma-A-weighted *F*_*o*_*-F*_*c*_, 1.9σ, black mesh). See also Table 1 and Figures S1, S2, and S3.

We solved the co-crystal structure of FluPol_C_ with a peptide comprising four repeats of the pS_5_-CTD heptad at 4.1 Å resolution (Table 1). From the difference electron density map, we are able to observe two distinct CTD-binding sites (Figure 1A). The better defined binding site we observe in FluPol_C_ (site 1) runs from the P3 C-terminal domain (equivalent to the PA C-terminal domain in FluPol_A/B_), close to the tips of helices α24 and α26, toward the P3 linker, between P3 C-terminal domain and PB1 helix α16 in the PB1 palm subdomain (Figures 1B, 1C, and S1). We were able to model 12 amino acid residues of the CTD with good geometry and consistent with the observed density. The modeled residues encompassed amino acids from three consecutive CTD repeats, T_4a_pS_5a_P_6a_S_7a_-Y_1b_S_2b_P_3b_T_4b_pS_5b_P_6b_S_7b_-Y_1c_ (Figure 1C). Our modeling was guided by a prominent hydrophobic pocket, which we used as an anchoring point and into which we placed the Y_1b_ of the peptide, in line with previously determined CTD complexes (Figure 1D) (Jasnovidova and Stefl, 2013, Lukarska et al., 2017). Overall, the CTD residues adopt an extended conformation across the binding surface, giving interaction areas of approximately 1,150 Å^2^ with P3 and 500 Å^2^ with PB1. In our model, the phosphate group of pS_5a_ lies in a pocket formed by PB1 residues H461 and R465 and P3 residue P237, whereas the other visible phosphoserine, pS_5b_, points out to the solvent (Figures 1C and 1E). Both tyrosines, Y_1b_ and Y_1c_, and threonine T_4b_ face FluPol_C_, with Y_1b_ lying close to P3 residues Y241, S661, F663, and PB1 W457; Y_1c_ near to P3 K657; and T_4b_ close to P3 residues N659 and K704 (Figures 1C and 1D). The second binding site we observe (site 2) is located within a groove formed by P3 β6 and the loop between P3 β11 and β12 (Figures 1F and S1). Because the electron density here was not of sufficient clarity for confident model building and refinement, we instead indicated the site by tracing a 10-residue poly-alanine chain. There was no visible electron density connecting the two binding sites, although they could potentially be bridged by a peptide encompassing four heptad repeats.

**Table 1:** Crystallographic Data Collection, Phasing, and Refinement Statistics

	FluPol_C_-pS_5_-CTD
**Diffraction Data**	

Space group	P 4_3_ 2_1_ 2
Cell dimensions (Å)	a = 185.7, b = 185.7, c = 597.6
	α = β = γ = 90
Wavelength (Å)	0.9795
Resolution range of data (last shell; Å)	131.38–4.14 (4.21–4.14)
Completeness (last shell; %)	93.6 (60.6)
CC1/2 (%)	99.8 (73.3)
R_merge_ (last shell; %)	20.9 (79.0)
I/σI (last shell)	7.9 (0.9)
Redundancy (last shell)	13.5 (2.1)

**Refinement**	

Reflections used in refinement (free)	55,611 (2,816)
R_work_ (%)	26.0
R_free_ (%)	29.6
Number of non-hydrogen atoms	35,232
Protein	35,228
Ligand	4

**Geometry and B factors**	

RMSD (bonds)	0.005
RMSD (angles)	0.81
Ramachandran favored (%)	97.37
Ramachandran outliers (%)	0.23
Average B factor	146.1
Protein	146.1
Ligand	139.5

RMSD, root-mean-square deviation.

The observed CTD binding sites in FluPol_C_ are distinct from all previously observed sites in FluPol_A_ and FluPol_B_ (Lukarska et al., 2017) (Figures 1G, S2, and S3). The location of site 1 in FluPol_C_ is different from site 1 in FluPol_A_ and FluPol_B_, and although spatially proximal, it is also distinct from site 2 reported in FluPol_B_. Site 2 in FluPol_B_ runs perpendicular to the interface between the PA_C_ domain and the PB2 627 domain; in our structure, the FluPol is found in a transcriptionally inactive conformation (Hengrung et al., 2015, Thierry et al., 2016), where the 627 domain lies level with the endonuclease domain, therefore making this position unavailable (Lukarska et al., 2017) (Figure 1G). The second binding site in FluPol_C_ (site 2) is in a position similar to site 2 of the FluPol_A_-CTD complex (Lukarska et al., 2017). However, the interaction details are likely to be different from FluPol_A_, because the CTD-binding residues of FluPol_A_ site 2 are not conserved in FluPol_C_.

### FluPol_C_ Amino Acid Residues Involved in CTD Binding Are Required for Viral Transcription

**Figure 2:**
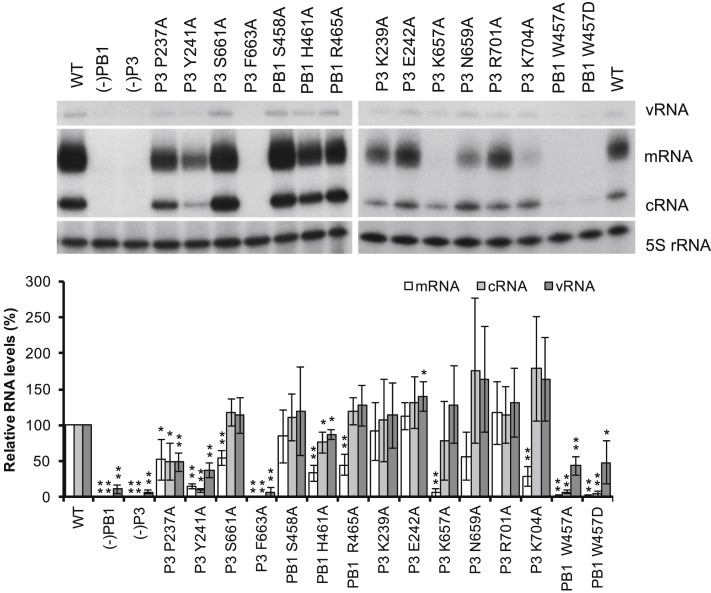
Effect of Mutations in CTD-Binding Site 1 on FluPol_C_ Function The effect of single-amino-acid mutations in CTD-binding site 1 was analyzed by RNP reconstitution assays. vRNA, mRNA, and cRNA levels were analyzed by primer extension and quantitated by phosphorimage analysis with 5S rRNA as a loading control. RNA levels generated by the wild-type (WT) polymerase were set to 100%. RNP reconstitutions without PB1 (−PB1) or P3 (−P3) served as negative controls. The mean of three independent experiments is shown with error bars representing SD. Asterisks indicate a significant difference from WT (two-tailed one-sample t test) as follows: ^∗^p < 0.05 and ^∗∗^p < 0.01. See also Figures S2 and S3.

To address whether the CTD-binding site identified in FluPol_C_ is functionally important, we mutated amino acid residues to alanines in the CTD-binding site 1 (Figures 1C, S2, and S3) and performed a minireplicon assay. We transfected 293T cells with wild-type or mutant polymerase and nucleoprotein expression plasmids, as well as a plasmid to express a vRNA template, to reconstitute vRNP complexes that produce viral mRNA as well as replication intermediate cRNA and genomic vRNA. The levels of these RNA products were quantified by primer extension analysis (Figure 2). Only low levels of vRNA, expressed from the transfected plasmid, were observed if the PB1 or P3 subunits were omitted from the transfection, in agreement with previous results (Hengrung et al., 2015). However, in the presence of a complete wild-type polymerase, we observed mRNA and cRNA, as well as a significant increase in vRNA levels, indicating that the viral polymerase transcribes and replicates the input vRNA. Comparing the RNA levels produced by the wild-type and mutant polymerases, we found that mutations K657A, N659A, S661A, and K704A in P3 specifically inhibited transcription (mRNA levels), but not replication (cRNA and vRNA levels). Furthermore, mutations of PB1 amino acids H461 or R465, which form the charged pocket accommodating pS_5a_ of the CTD, also resulted in specific inhibition of transcription but in no effect or only a small effect on replication. The phenotype of these mutants is fully consistent with the idea that CTD binding is required for viral transcription, as this interaction facilitates access to 5′ caps of nascent Pol II transcripts. Mutations at amino acids P237, Y241, and F663 in P3 and W457 in PB1 affected both transcription and replication, possibly due to their position at the PB1-P3 interface, which might cause them to interfere with heterotrimer assembly. In contrast, mutations K239A, E242A, and R701A in P3 and S458A in PB1 had no major effect on polymerase activity, indicating that these amino acid residues do not contribute critical interactions to CTD binding.

### PB2 627 and NLS Domains of FluPol_C_ Are Involved in CTD Binding

**Figure 3:**
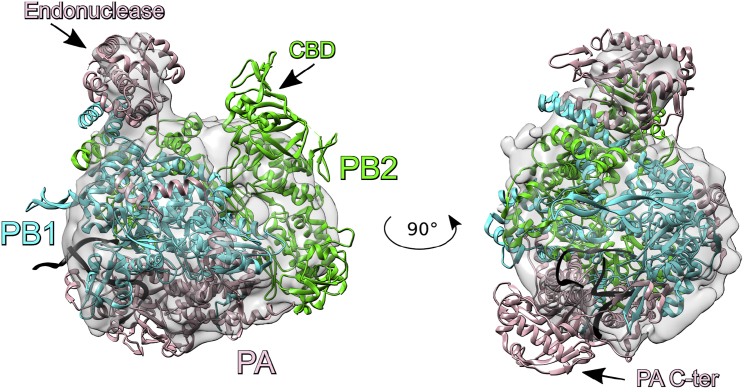
Cryo-EM Analysis of FluPol_C_ Bound to vRNA Promoter Structure of the vRNA promoter-bound FluPol_B_ in a transcription pre-initiation conformation (PDB: 4WSA) fit into the cryo-EM density map of FluPol_C_ bound to vRNA promoter. See also Figure S4.

The structure of FluPol_C_ in complex with the Pol II CTD has been obtained in a transcriptionally inactive conformation (Figure 1A). However, CTD-bound FluPol is proposed to carry out cap snatching and therefore must be present in its transcription pre-initiation state. To investigate the conformational states that FluPol_C_ can assume, we collected a cryo-EM dataset of vRNA promoter-bound FluPol_C_, which resulted in a 9.8-Å reconstruction (Figure 3). We attempted fitting both the transcription pre-initiation conformation of FluPol_B_ and the transcriptionally inactive apo form of FluPol_C_ into the cryo-EM density map (Figures 3 and S4A). Overall, the transcription pre-initiation conformation of FluPol_B_ produced a significantly better fit, with clear density for the PA endonuclease domain. In agreement with previous observations that the PB2 C-terminal domains are extremely flexible (Hengrung et al., 2015, Reich et al., 2014, Thierry et al., 2016), the corresponding regions of PB2 in our reconstruction were not resolved, presumably due to dynamic motion. This suggests that, when bound to the vRNA promoter, the polymerase exists as a heterogeneous mixture of conformations, with PB2 undergoing large shifts, which is consistent with what has been observed for vRNA promoter-bound FluPol_B_, where large regions of PB2 electron density are missing in the crystal structure (Reich et al., 2014). Part of the PA C-terminal domain also fits outside the density, but this region correlates with an area of poor resolution, likely due to particles acquiring some preferential orientations in vitreous ice (Figures S4A and S4B). Our cryo-EM data support a model where vRNA promoter-bound FluPol_C_ is dynamic in solution. Thus, whereas FluPol_C_ can indeed acquire a transcription pre-initiation conformation, this is likely only transient. The presence of promoter vRNA alone therefore appears to be insufficient to stabilize the polymerase in solution in the transcription pre-initiation conformation.

**Figure 4:**
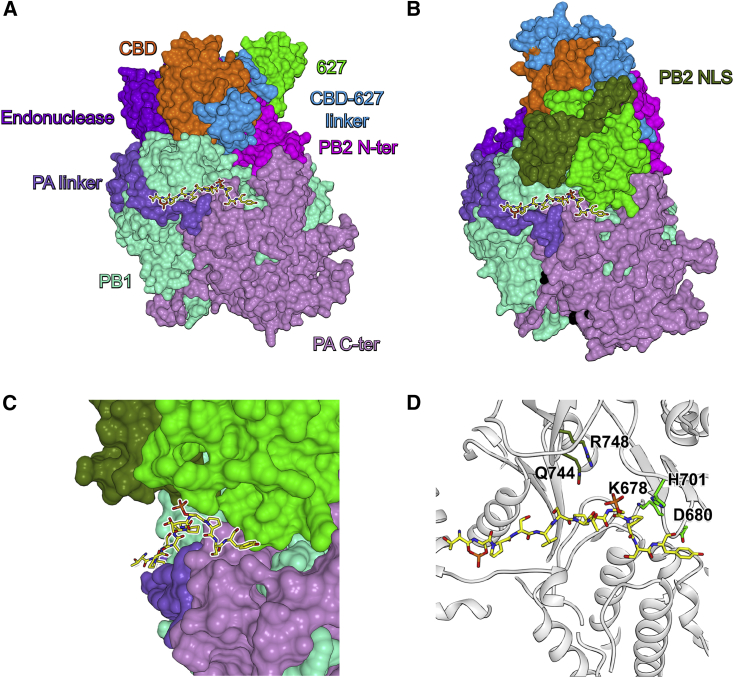
Modeled FluPol_C_ in the Transcription Pre-initiation Conformation Bound to Pol II CTD Peptide (A) Surface representation of pS_5_-CTD-bound FluPol_C_ crystal structure in a transcriptionally inactive conformation with highlighted subunit domain arrangement. pS_5_-CTD is shown in yellow. (B) Surface representation of modeled pS_5_-CTD-bound FluPol_C_ in a transcription pre-initiation conformation with highlighted subunit domain arrangement. The vRNA promoter is shown in black. (C) Detailed view of the PB2 627 and NLS domains packing above the pS_5_-CTD peptide in the transcription pre-initiation conformation of FluPol_C_. (D) Detailed view of the pS_5_-CTD peptide interaction at site 1 in the modeled transcription pre-initiation conformation of FluPol_C_ with key amino acid residues in the PB2 627 and NLS domains highlighted. See also Figure S5.

**Figure 5:**
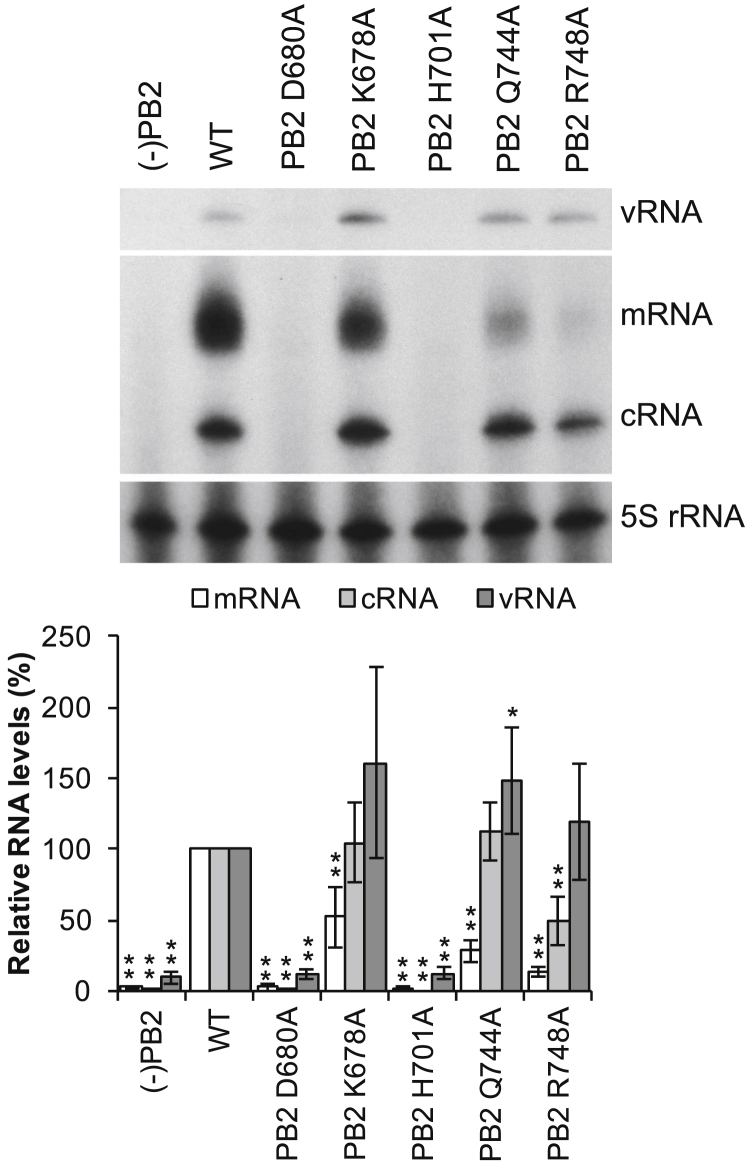
Effect of Mutations in the PB2 627 and NLS Domains on FluPol_C_ Function The effect of single-amino-acid mutations in the PB2 627 and NLS domains proximal to CTD-binding site 1 was analyzed by RNP reconstitution assays. vRNA, mRNA, and cRNA levels were analyzed by primer extension and quantitated by phosphorimage analysis with 5S rRNA as a loading control. RNA levels generated by the WT polymerase were set to 100%. RNP reconstitutions without PB2 (−PB2) served as negative controls. The mean of five independent experiments is shown with error bars representing SD. Asterisks indicate a significant difference from WT (two-tailed one-sample t test) as follows: ^∗^p < 0.05 and ^∗∗^p < 0.01. See also Figure S5.

Next, we modeled FluPol_C_ in a transcription pre-initiation conformation, based on the domain arrangement observed in the cryo-EM structure above and the available FluPol_A_ and FluPol_B_ structures (Figure 4) (Pflug et al., 2014, Reich et al., 2014). This revealed that, in the transcription pre-initiation conformation, the PB2 627 and NLS domains would lie close to CTD-binding site 1 and could potentially directly interact with the CTD peptide, hence contributing to CTD binding (Figures 4B and 4C). To address this experimentally, we mutated five surface-exposed amino acid residues of the FluPol_C_ PB2 627 and NLS domains pointing toward the CTD in our model (Figure 4D). Using vRNP reconstitution assays, as described above, we found that mutations D680A and H710A abolished both transcription and replication, whereas K678A, Q744A, and R748A specifically reduced transcription without having a major effect on vRNA replication (Figures 5 and S5). These results are fully consistent with the 627 and NLS domains of PB2 binding the CTD of Pol II, stabilizing FluPol in the transcription pre-initiation conformation.

### Pol II CTD Peptide Enhances FluPol_C_ Transcriptional Activity *In Vitro*

**Figure 6:**
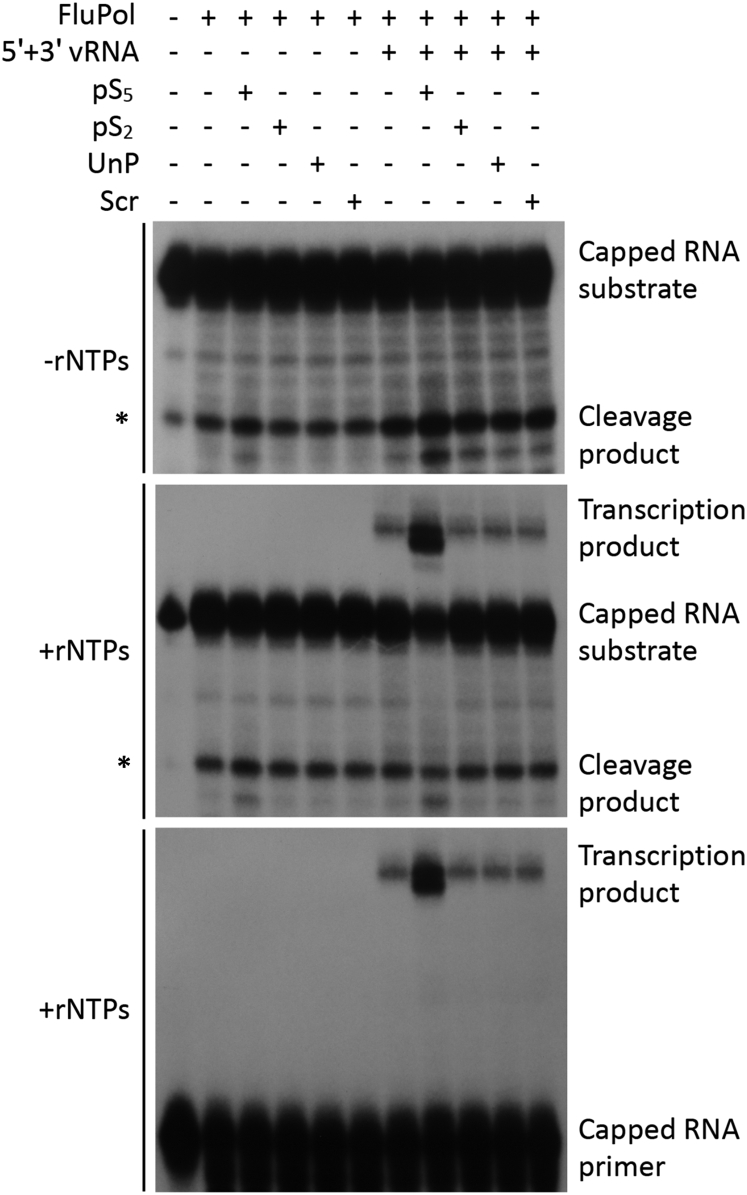
Effect of Pol II CTD Peptides on FluPol_C_ Endonuclease and Transcriptional Activity *In vitro* endonuclease and cap-dependent transcription initiation assays were performed using purified FluPol_C_ in the presence of pS_5_, pS_2_, un-phosphorylated (UnP), or scrambled (Scr) Pol II CTD peptides. Assays were carried out in the absence or presence of 5′ and 3′ vRNA promoter RNAs. The absence or presence of rNTP substrates is indicated. Top panel: capped RNA cleavage assay is shown; middle panel: capped RNA cleavage and transcription initiation assay are shown; bottom panel: cap-dependent transcription initiation assay is shown. The position of a non-specific cleavage product that partially overlaps with the viral polymerase-specific cleavage products is indicated by a star (^∗^). See also Figure S6.

FluPol is inherently flexible, and in solution, it explores different conformational distributions depending on RNA binding (Hengrung et al., 2015, Thierry et al., 2016). However, it remains unclear how the different conformational states are regulated in response to RNA binding and possibly also in response to interactions with host factors. Our data above suggest that binding of FluPol_C_ to the Pol II CTD might stabilize the transcription pre-initiation conformation through interactions between the CTD and the PB2 627 and NLS domains. Therefore, we sought to determine whether the addition of pS_5_-CTD peptide affected the transcriptional activity of FluPol *in vitro*, which is dependent on FluPol, assuming the cap-snatching competent, transcription pre-initiation conformation. We found that the cap-dependent endonuclease activity of vRNA-bound FluPol_C_ was enhanced in the presence of the pS_5_-CTD peptide, resulting in the generation of a capped RNA primer from a 20-nt-long radiolabeled capped RNA (Figure 6, top panel). In the presence of ribonucleoside triphosphates (rNTPs), the resulting capped RNA was used by FluPol_C_ as a primer to initiate transcription, leading to the production of a capped transcription product (Figure 6, middle panel). Addition of Ser2-phosphorylated (pS_2_), unphosphorylated (UnP), or scrambled (Scr) peptide had no effect on the cleavage of a 20-nt-long radiolabeled capped RNA, in agreement with pS_5_ being required for the interaction with FluPol (Engelhardt et al., 2005, Lukarska et al., 2017, Martínez-Alonso et al., 2016). A specific capped RNA cleavage product was also observed in the absence of vRNA (Figure 6, top and middle panels), indicating that CTD alone can stabilize the transcription pre-initiation conformation of the polymerase to some extent. However, vRNA binding resulted in increased cleavage, indicating that vRNA binding is also important for the conformational rearrangement as suggested by previous studies (Hengrung et al., 2015, Thierry et al., 2016). In order to determine whether only the endonuclease activity or also transcription initiation is enhanced in presence of pS_5_-CTD peptide, we performed an *in vitro* transcription assay in which FluPol_C_ was provided with a radiolabeled 11-nt-long capped RNA primer that can be extended by the polymerase directly without prior cleavage. Addition of pS_5_-CTD also increased transcription initiation (Figure 6, bottom panel). Therefore, both the cleavage of capped RNA and capped primer-dependent transcription initiation are enhanced when pS_5_-CTD peptide is present. These results are fully consistent with a model in which FluPol_C_ in solution is highly dynamic, flipping between a number of conformations, even when bound to promoter vRNA, but is stabilized in the transcription pre-initiation conformation upon binding to Pol II pS_5_-CTD.

## Discussion

In this study, we identify the Pol II CTD binding site in FluPol_C_ and propose a model for the transcriptional activation of FluPol through binding to host Pol II. Despite the relatively low sequence conservation found between FluPol_A_, FluPol_B_, and FluPol_C_, they are all able to bind pS_5_-CTD, highlighting that binding to Pol II is essential for transcriptional polymerase activity. As all influenza viruses rely on cap snatching to produce mRNA, association with the Pol II CTD provides the viral polymerase with direct access to host-capped RNA. Furthermore, interacting specifically with the pS_5_ form of the CTD ensures FluPol_C_ is present during the brief initial pausing event of Pol II while nascent Pol II transcripts are capped, presumably prior to the capped RNAs being bound by the host nuclear cap-binding complex. Interestingly, the binding sites identified in FluPol_C_ are distinct from those observed in FluPol_A_ and FluPol_B_, illustrating the variety of solutions to CTD binding that the different influenza types have evolved. Site 2 on our FluPol_C_ structure lies at a similar position as the site 2 identified in FluPol_A_, but the lack of conserved amino acids in that region implies that the interaction must involve a different set of residues. The site 1 identified on FluPol_C_ between the P3 C terminus and PB1 is, yet again, entirely distinct despite its somewhat comparable location when compared to the second site observed in FluPol_B_. Our work also shows, in agreement with previous data (Lukarska et al., 2017), that the FluPol can bind multiple, non-adjacent repeats of the Pol II CTD. The presence of multiple binding sites, which are quite distant from each other, suggests that the Pol II CTD could be partially wrapping around the FluPol, forming multiple, low-affinity interactions but with higher avidity, as is frequently the case in disordered proteins (Dunker et al., 2002, Zhou, 2012).

**Figure 7:**
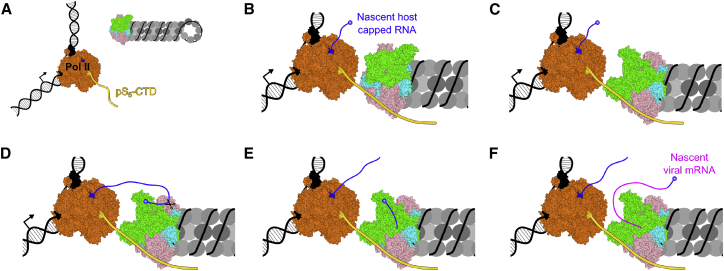
Model for Viral Transcription Activation Induced by Binding to Pol II CTD (A) Prior to association with pS_5_-CTD of Pol II, multiple conformations of vRNP-associated FluPol exist in a dynamic equilibrium. However, most FluPol is likely present in a transcriptionally inactive conformation, in agreement with its low transcriptional activity. (B) FluPol binds to the pS_5_-CTD of Pol II. (C) Binding to the CTD stabilizes FluPol in the transcription-competent conformation as the PB2 627 and NLS domains interact with pS_5_-CTD. (D) In the transcription pre-initiation conformation, with the PB2 cap-binding and PA endonuclease domains reconfigured for cap snatching, FluPol binds the nascent-capped RNA produced by Pol II. (E) Cap snatching occurs, resulting in a short, capped RNA primer, which inserts into the FluPol active site. (F) Transcription initiation takes place, and the capped RNA primer is elongated by FluPol to produce viral mRNA.

Our structure of FluPol_C_ in complex with a Pol II CTD peptide offers insights into the stabilization of the transcription-competent conformation of the polymerase: site 1 is positioned at an ideal location to interact with the PB2 627 and NLS domains, which, in the transcription pre-initiation conformation, would pack against the CTD peptide. The PB2 627 domain of FluPol_A_ has been attributed a role in host range restriction of influenza viruses, although its role in polymerase function remains still poorly defined (Long et al., 2016, Nilsson et al., 2017). Our work suggests that the PB2 627 and NLS domains are important for viral transcription by interacting with the Pol II CTD, which contributes to the stabilization of the transcription pre-initiation conformation of the polymerase (Figure 7). This is consistent with previous observations that the PB2 627 domain is essential for transcription *in vivo*, although it is not required for cap-dependent transcription initiation *in vitro* (Nilsson et al., 2017). Thus, the PB2 627 domain is not only important for RNA genome replication by mediating the recruitment of a polymerase to nascent viral RNA as proposed previously (Nilsson et al., 2017), but it also plays a role in transcription, highlighting the multifunctional nature of the influenza virus polymerase. The finding that the CTD interacts with the 627 domain of FluPol_B_ (Lukarska et al., 2017) suggests that the PB2 627 domain could also play a similar role in stabilizing the transcription pre-initiation FluPol conformation in other influenza types. Indeed, we observed a significant increase in capped-RNA-primed transcriptional activity of FluPol_A_ in the presence of pS_5_ compared to when pS_2,_ UnP, or Scr CTD peptides were added or no CTD peptide was present (Figure S6). These results suggest that the transcriptase conformation of FluPol_A_ is also stabilized upon binding to Pol II CTD. Stabilization of the transcription-competent conformation of FluPol through binding to the CTD of Pol II provides an elegant regulatory mechanism for the activation of the cap-snatching, and thus transcriptional, activity of the influenza virus polymerase and could represent a switch between the transcriptase and replicase functions of FluPol. However, further work will need to be carried out to determine exactly how the polymerases of different influenza virus types in the context of vRNPs interact with the host transcriptional machinery.

In summary, we have identified two Pol II CTD binding sites on FluPol_C_, one in a position similar, but not identical, to site 2 of FluPol_A_ and another in a completely distinct position at the interface of the P3 C-terminal domain and the PB1 palm subdomain. Despite the presence of multiple binding sites, single point mutations at site 1 result in a dramatic impairment of transcription, which highlights the dependency of FluPol on optimal association with the Pol II CTD. This makes the FluPol-Pol II CTD interaction an attractive target for anti-viral small molecule inhibitor development.

## STAR★Methods

### Key Resources Table

**Table:** 

REAGENT or RESOURCE	SOURCE	IDENTIFIER
**Bacterial and Virus Strains**		

Recombinant baculovirus expressing FluPol_C_ PB1, P3 and PB2-protein A	Bieniossek et al., 2012, Hengrung et al., 2015	N/A
Recombinant baculovirus expressing FluPol_A_ PB1, P3 and PB2-protein A	Bieniossek et al., 2012, York et al., 2013	N/A

**Chemicals, Peptides, and Recombinant Proteins**		

AcTEV protease	Thermo Fisher Scientific	Cat# 12575015
SuperScript III	Thermo Fisher Scientific	Cat# 18080093
pS_5_-CTD: Biotin-PEG-YSPTS^∗^PSYSPTS^∗^ PSYSPTS^∗^PSYSPTS^∗^PS	Cambridge Peptides; Martínez-Alonso et al., 2016	N/A
pS_2_-CTD: Biotin-PEG-YS^∗^PTSPSYS^∗^PTSPSYS^∗^ PTSPSYS^∗^PTSPS	Cambridge Peptides; Martínez-Alonso et al., 2016	N/A
UnP-CTD: Biotin-PEG-YSPTSPSYSPTSPSYS PTSPSYSPTSPS	Cambridge Peptides; Martínez-Alonso et al., 2016	N/A
Scr-CTD: Biotin-PEG-PSSSTPSSYTPSPSSS PTSYSPYYTSPP	Cambridge Peptides; Martínez-Alonso et al., 2016	N/A
IgG Sepharose 6 Fast Flow	GE Healthcare	Cat# 17096901
Hi-Load Superdex 200 16/60	GE Healthcare	Cat# 45-002-490
Lipofectamine 2000	Thermo Fisher Scientific	Cat# 11668027
TRI Reagent	Sigma-Aldrich	Cat# T9424
[α-^32^P] GTP	PerkinElmer	Cat# BLU006H250UC
Vaccinia capping system	New England Biolabs	Cat# M2080S

**Deposited Data**		

Coordinates of pS_5_-CTD bound FluPol_C_	This paper	PDB: 6F5O
Cryo-EM map of promoter vRNA bound FluPol_C_	This paper	EMDB: EMD-4190
Imaging data deposited in Mendeley	This paper	https://doi.org/10.17632/tswbw8d2h7.1

**Experimental Models: Cell Lines**		

HEK293T cells	Dunn School Cell Bank	N/A
Sf9 insect cells	Dunn School Cell Bank	N/A

**Oligonucleotides**		

vRNA RT primer 5′-CGCAAGGCGACAA GGTGCTGA-3′	Fodor et al., 2002	N/A
m/cRNA RT primer 5′-ATGTTCTTTACGA TGCGATTGGG-3′	Fodor et al., 2002	N/A
5S rRNA RT primer 5′-ACCCTGCTTAGCTT CCGAGA-3′	Hara et al., 2006	N/A
RNA for capping (20 nt) 5′-ppAAUCUAUAAUAG CAUUAUCC-3′	Chemgenes; Hengrung et al., 2015	N/A
RNA for capping (11 nt) 5′-ppGAAUACUCAAG-3′	Chemgenes; Hengrung et al., 2015	N/A
5′ FluPol_C_ vRNA promoter RNA 5′-AGCAGUAGCAA GGGG-3′	Dharmacon; Hengrung et al., 2015	N/A
3′ FluPol_C_ vRNA promoter RNA 5′-CCCCUGCUU CUGCU-3′	Dharmacon; Hengrung et al., 2015	N/A
5′ FluPol_A_ vRNA promoter RNA 5′- AGUAGAAAC AAGGCC-3′	Dharmacon; Fodor et al., 2002	N/A
3′ FluPol_A_ vRNA promoter RNA 5′- GGCCUGCU UUUGCU-3′	Dharmacon; Fodor et al., 2002	N/A

**Recombinant DNA**		

pHMG PB1	Donated by Nadia Naffakh; Crescenzo-Chaigne et al., 1999	N/A
pHMG PB2	Donated by Nadia Naffakh; Crescenzo-Chaigne et al., 1999	N/A
pHMG PA	Donated by Nadia Naffakh; Crescenzo-Chaigne et al., 1999	N/A
pHMG NP	Donated by Nadia Naffakh; Crescenzo-Chaigne et al., 1999	N/A
pC/PRCAT	Donated by Nadia Naffakh; Crescenzo-Chaigne et al., 1999	N/A

**Software and Algorithms**		

Chimera 1.10.2	Pettersen et al., 2004	https://www.cgl.ucsf.edu/chimera/download.html
ResMap	Kucukelbir et al., 2014	http://resmap.sourceforge.net/
RELION (VLION)	Scheres, 2012	https://www2.mrc-lmb.cam.ac.uk/relion/index.php/Download_%26_install
EMAN2	Tang et al., 2007	http://blake.bcm.edu/emanwiki/EMAN2
Motioncor2	Zheng et al., 2017	http://msg.ucsf.edu/em/software/motioncor2.html
CryoSPARC	Punjani et al., 2017	https://v2.cryosparc.com/
GCTF	Zhang, 2016	https://www.mrc-lmb.cam.ac.uk/kzhang/Gctf/
Xia2	Winter et al., 2013	https://xia2.github.io/
PHASER	McCoy et al., 2007	http://www.phaser.cimr.cam.ac.uk/index.php/Phaser_Crystallographic_Software
COOT	Emsley and Cowtan, 2004	https://www2.mrc-lmb.cam.ac.uk/personal/pemsley/coot/
PHENIX	Adams et al., 2010	https://www.phenix-online.org/
SWISS-MODEL Oligomer server	Biasini et al., 2014	https://swissmodel.expasy.org/
Aida Image Analyzer (3.27.001)	Elysia Raytest	N/A

### Contact for Reagent and Resource Sharing

Further information and requests for resources and reagents should be directed to and will be fulfilled by the Lead Contact, Ervin Fodor (ervin.fodor@path.ox.ac.uk).

### Experimental Model and Subject Details

#### HEK293T Cells

Human Embryonic Kidney 293 T cells (female cell line) were grown in Dulbecco’s Modified Eagle Medium (Sigma-Aldrich) supplemented with 10% fetal calf serum (Sigma-Aldrich). Cells were maintained at 37°C, 5% CO_2_. Cells were trypsinised with TrypLE Express Enzyme (GIBCO).

#### Sf9 Insect Cells

*Spodoptera frugiperda* 9 insect cells (female cell line) were grown in Insect-XPRESS protein-free insect cell medium (Lonza) supplemented with 1x PenStrep (GIBCO). Cell density was maintained between 0.5-2 million cells/ml. Cells were maintained at 27°C, shaking at 110 rpm.

The cell lines used have not been authenticated.

### Method Details

#### Expression and Purification of FluPol_A_ and FluPol_C_

Influenza A/NT/60/68 (H3N2) and C/Johannesburg/1/1966 virus polymerases were expressed using a recombinant baculovirus and purified from insect cells as described (Bieniossek et al., 2012, Hengrung et al., 2015, York et al., 2013). The heterotrimeric complex was purified from 2 L Sf9 insect cells infected with a baculovirus encoding the three polymerase genes, where the PB2 gene had a C-terminal protein A tag. Cells were grown to a density of 2 million cells/ml and infected with 6 mL V_2_ virus stock per liter of culture. The cells were incubated for 72 h before harvesting by centrifugation at 800 g for 15 min at 4°C. The cell pellet was resuspended in 50 mL lysis buffer (25 mM HEPES pH 7.5, 500 mM NaCl, 10% glycerol, 0.05% OTG, 1x Complete EDTA-free protease inhibitors (Roche)). The cells were lysed by sonication and then centrifuged at 35000 g for 45 min at 4°C. The supernatant was applied to 4 mL equilibrated IgG Sepharose beads and incubated for 3 h. The beads were washed three times with 25 mL wash buffer (25 mM HEPES pH 7.5, 500 mM NaCl, 10% glycerol, 0.05% OTG). For every wash, the beads were incubated for 10 min, rotating at 4°C and then centrifuged at 1000 g for 1 min. The complex was released by incubation with 40 μL TEV protease in 12 mL cleavage buffer (25 mM HEPES pH 7.5, 500 mM NaCl, 10% glycerol, 0.05% OTG, 2.5 mM reduced glutathione) overnight at 4°C. The beads were removed by centrifugation at 4000 g for 5 min. The complex was then further purified by size exclusion chromatography on a Hi-Load Superdex 200 16/60 gel filtration column (GE Healthcare) connected to an Äkta chromatography system (GE Healthcare) at 4°C in gel filtration buffer (25 mM HEPES pH 7.5, 500 mM NaCl, 10 mM MgCl_2_ and 10% (v/v) glycerol). The peak fractions were pooled and concentrated to the desired concentration using an Amicon Ultra centrifugal filter unit (Merck Millipore, NMWL 100 kDa).

#### Crystallization and Structure Determination

For crystallization, a 5 mg/ml solution of FluPol_C_ in 25 mM HEPES:NaOH pH 7.5, 0.5 M NaCl, 10 mM MgCl_2_ and 10% (v/v) glycerol was supplemented with 10% (v/v) pS_5_-CTD peptide (2.7 mM in DMSO) and 0.5 mM TCEP. This was used for sitting-drop vapor-diffusion crystallization experiments at 20°C. Commercial sparse matrix screens were employed to find adequate crystallization conditions including Morpheus (Molecular Dimensions), PEGRx, MembFac, Hampton Natrix Screen and Hampton Crystal Screen Cryo (Hampton Research), Nuc-Pro (Jena Bioscience) and Emerald Wizard I/II (Emerald BioSystems). The best crystal grew from a drop equilibrated against 70% (v/v) Morpheus G2 (Molecular Dimensions) and 7.5% (v/v) glycerol, set up with an initial protein:precipitant ratio of 2:1. This was cryo-protected using 25% (v/v) glycerol in crystallization buffer and then flash cooled in liquid nitrogen. The crystal was exposed at the Diamond Light Source (Didcot, UK), on beamline I04, equipped with a Pilatus 6M detector and using a wavelength of 0.9763 Å.

Reduction of X-ray diffraction data was carried out automatically using Xia2 (Kabsch, 2010, Winter et al., 2013). The scaled and merged data were then anisotropy-corrected with the STARANISO server (http://staraniso.globalphasing.org/). The structure of FluPol_C_ in complex with the pS_5_-CTD peptide was solved by molecular replacement in PHASER (McCoy et al., 2007) utilizing the published apo-structure (PDB: 5D98) as search model. We subjected the structure to multiple rounds of manual building in COOT (Emsley and Cowtan, 2004) and refinement in PHENIX (Adams et al., 2010) utilizing TLS-parameters, Ramachandran and secondary structure restraints, and two-fold NCS-restraints. The structure was validated using the wwPDB Validation Service (https://validate-rcsb-1.wwpdb.org/). Data collection and refinement statistics are shown in Table 1. Note that the given refinement statistics refer to the anisotropy corrected data.

#### Minireplicon Assays Using RNP Reconstitutions and Primer Extension

Plasmids to express the three FluPol_C_ subunits (pHMG-PB1, pHMG-PB2 and pHMG-P3), nucleoprotein (pHMG-NP) and a plasmid expressing a negative-sense CAT reporter gene flanked by the terminal non-coding sequences of the influenza C/Johannesburg/1/1966 virus NS gene segment (pC/PRCAT) (all kind gifts of Nadia Naffakh, Pasteur Institute Paris), were transfected into HEK293T cells using Lipofectamine 2000 (Invitrogen) (Crescenzo-Chaigne et al., 1999). Total RNA was isolated 30 hr post-transfection by phenol-choroform extraction using Tri Reagent (Invitrogen) and analyzed by reverse transcription with ^32^P-labeled primers 5′-CGCAAGGCGACAAGGTGCTGA-3′ (for detection of vRNA, yielding a 127-nucleotide product) and 5′-ATGTTCTTTACGATGCGATTGGG-3′ (for detection of mRNA and cRNA, yielding 98–102 nucleotide and 89 nucleotide products, respectively) (Fodor et al., 2002). A ^32^P-labeled primer 5′-ACCCTGCTTAGCTTCCGAGA-3′ was used to detect 5S rRNA as an internal loading control, for which the expected size of the product was 62 nt (Hara et al., 2006). Input levels of vRNA produced from the pC/PRCAT plasmid were determined by including an RNP reconstitution where one of the plasmids encoding a polymerase subunit was omitted. Products were analyzed by PAGE on 6% acrylamide:bisacrylamide (19:1) gels containing 7 M urea, and detected by autoradiography for generating images for presenting unprocessed data. Quantitation of the data was carried out by exposing the gels directly to phosphor screen imaging plates (Fujifilm), which were scanned on a Fujifilm FLA-5000 scanner. Aida Image Analyzer software (Raytest) was used to determine band intensities.

#### *In Vitro* FluPol Activity Assays

Synthetic RNAs 5′-ppAAUCUAUAAUAGCAUUAUCC-3′ and 5′-ppGAAUACUCAAG-3′ (Chemgenes) were capped and radiolabelled by incubation with [α-^32^P] GTP, vaccinia virus capping enzyme (NEB) and 2′-*O*-methyltransferase (NEB), following the manufacturer’s instructions. For endonuclease activity assays, 0.5 μL capped RNA was mixed with 1.5 μL purified polymerase (at a final concentration of 142 nM) in 7.5 mM MgCl_2_, 2 mM DTT, and 1 U/μl RNasin (Promega), with or without 0.5 μM 5′ vRNA promoter (5′-AGCAGUAGCAAGGGG-3′) and 0.5 μM 3′ vRNA promoter (5′-CCCCUGCUUCUGCU-3′), in a total reaction volume of 4 μl. For capped RNA primed transcription assays, ATP, UTP, CTP and GTP were added at a final concentration of 0.5 mM each. Pol II CTD peptides (Martínez-Alonso et al., 2016) were added at a final concentration of 1.42 μM where indicated. The reactions were incubated at 30°C for 1 hr. RNA products were analyzed by PAGE on 18% acrylamide:bisacrylamide (19:1) gels containing 7 M urea and detected by autoradiography.

#### Cryo-electron Microscopy

Purified FluPol_C_ was mixed with a 1.2 fold molar excess of 5′ and 3′ promoter vRNAs (5′ vRNA: 5′-AGCAGUAGCAAGGGG-3′; 3′ vRNA: 5′-CCCCUGCUUCUGCU-3′) prior to grid preparation. 3 μL of vRNA promoter-bound FluPol_C_ at 0.5 mg/ml was placed on glow discharged carbon-coated (40 nm film) copper C-flat grids (Protochips) with 2 μm holes and 1.2 μm spacing before blotting for 3 s and flash-freezing in liquid ethane. Grids were prepared using a Vitrobot mark IV (FEI) at 80%–100% humidity. 1822 micrographs were collected on a 300 keV Polara microscope (FEI) fitted with a K2 direct electron detector (Gatan) at magnification 37037x (pixel size 1.35 Å), defocus range −3.5 to −2 μm. A total dose of 46 e^−^/Å^2^ was applied over 22 frames, with a total exposure time of 8.8 s (dose rate 5.23 e^−^/Å^2^/s). Beam-induced drift was corrected using MotionCor2 (Zheng et al., 2017) and CTF correction was carried out with GCTF (Zhang, 2016). Particle picking was carried out in EMAN2 (Tang et al., 2007). All further data processing was carried out using RELION (VLION) (Scheres, 2012). A total of 48648 particles were extracted of which 15789 remained after 2D classification. Most 2D classes contained particles in two orientations. Particle numbers in different views were therefore balanced to obtain a better distribution of orientations, which resulted in a final set of 9159 particles for 3D reconstruction. 3D reconstruction and refinement were carried out using the apo FluPol_C_ structure (PDB: 5D98) filtered to 40 Å as an initial reference model. Local map resolution was calculated using ResMap (Kucukelbir et al., 2014). Fitting of atomic models into the cryo-EM map was carried out using Chimera (Pettersen et al., 2004).

#### Modeling of pS_5_-CTD Bound FluPol_C_ in the Transcription Pre-initiation Conformation

A model was generated using the SWISS-MODEL Oligomer server (https://swissmodel.expasy.org/) (Biasini et al., 2014), utilizing the deposited bat FluPol_A_ (PDB: 5M3H) as a template. The pS_5_-CTD was placed onto the model according to our crystal structure. Finally, the geometry was optimized using the program phenix.geometry_minimization (Adams et al., 2010), yielding good Ramachandran statistics (95.66% favored, 0.68% outliers) and a MolProbity score of 2.0 (Chen et al., 2010).

### Quantification and Statistical Analysis

Statistical parameters are stated in figure legends and under the appropriate Method details section.

#### Minireplicon Assays

Minireplicon assays (Figures 2 and 5) were performed three (n = 3 for Figure 2) or five times (n = 5 for Figure 5). The intensity of the bands corresponding to the different RNA species were quantitated with Aida Image Analyzer software (Raytest). 5S rRNA signal was used as an internal loading control and therefore all band intensities were normalized against the corresponding 5S rRNA signal. Wild-type band intensities were set to represent 100%. The mean of three (Figure 2) or five (Figure 5) experiments was used and standard deviations calculated (MS Excel). A two-tailed one-sample t test was employed to determine significant differences between RNA levels produced by wild-type and mutant polymerases. p values below 0.05 (^∗^, p < 0.05) were considered significant.

#### Cryo-EM

The statistical analyses for map refinement were integral parts of the Relion software package (Scheres, 2012). Local map resolution (Figure S4) was calculated using ResMap (Kucukelbir et al., 2014) with the following parameters: min. resolution 7, max. resolution 15, step resolution: 0.25, voxel size 1.35. Angular distribution of particle orientations was calculated with the cryoSPARC software package (Punjani et al., 2017).

#### *In Vitro* Polymerase Activity Assays

*In vitro* polymerase activity assays (Figure S6) were performed three times (n = 3). The intensity of the bands corresponding to the transcription product RNA species were quantitated with Aida Image Analyzer software (Raytest). Band intensities corresponding to the reaction in absence of CTD peptide, in presence of promoter vRNA, were set to represent 100% activity. The mean of three experiments was plotted and standard deviations calculated (MS Excel). A two-tailed one-sample t test was employed to determine significant differences between reactions in presence versus absence of CTD peptides. p values below 0.01 (^∗∗^p < 0.01) were considered significant.

### Data and Software Availability

The accession numbers for the data reported in this paper are PDB: 6F5O and EMDB: EMD-4190.

## Supplemental Information

Document S1. Figures S1–S6

Document S2. Article plus Supplemental Information
