# A Mechanism for the Activation of the Influenza Virus Transcriptase

**DOI:** 10.1016/j.molcel.2018.10.005

**Published:** 2018-10-18

**Authors:** Itziar Serna Martin, Narin Hengrung, Max Renner, Jane Sharps, Mónica Martínez-Alonso, Simonas Masiulis, Jonathan M. Grimes, Ervin Fodor

(Molecular Cell *70*, 1101–1110.e1–e4; June 21, 2018)

In the originally published article, one of the PDB ID numbers shown in the Key Resources Table and in the STAR Methods under Data and Software Availability was incorrectly specified. In addition, an important PDB ID was missing. PDB: 6F5O contains coordinates of FluPol_B_ fitted into the cryo-EM map of promoter vRNA bound FluPol_C_. Coordinates of pS_5_-CTD bound FluPol_C_ can be found at PDB: 6F5P. Both of these changes have been made to the article online, and the authors apologize for any confusion.

