# Supplementary material for: A Mechanism for the Activation of the Influenza Virus Transcriptase
Source: Mol Cell. 2018 Jun 21;70(6):1101–1110.e4. doi: 10.1016/j.molcel.2018.05.011 (PMC6024077; doi:10.1016/j.molcel.2018.05.011)
Supplement: Document S1. Figures S1–S6 [file mmc1.pdf]

Molecular Cell, Volume *70*

Supplemental Information

A Mechanism for the Activation of the Influenza Virus Transcriptase

Itziar Serna Martin, Narin Hengrung, Max Renner, Jane Sharps, Mónica Martínez-Alonso, Simonas Masiulis, Jonathan M. Grimes, and Ervin Fodor

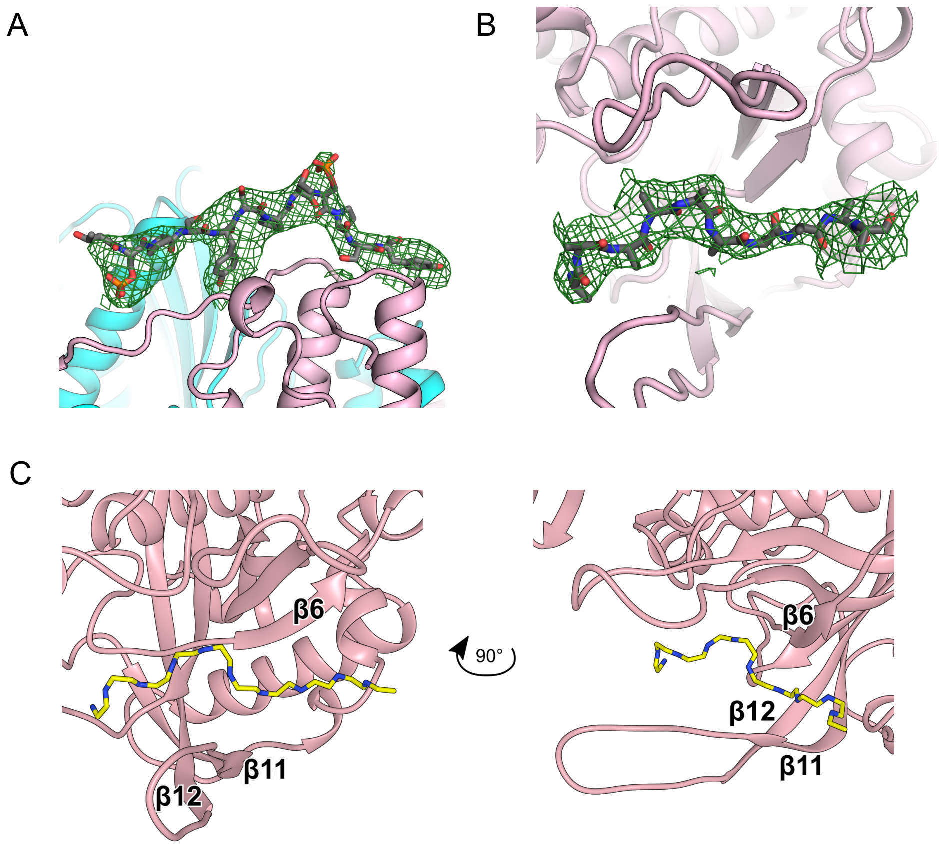

Figure S1. Detailed view of pS$_5$-CTD binding sites on FluPol$_C$.

(A) Detailed view of the pS$_5$-CTD peptide inside the omit electron density map (Sigma-A weighted F_o-F_c, 2.5σ, green mesh) for pS$_5$-CTD binding site 1.

(B) Same as (A) for pS$_5$-CTD binding site 2.

(C) Ribbon representation of the pS$_5$-CTD binding site 2 in the P3 C-ter domain involving a groove formed by P3 β6 and the loop between P3 β11 and β12. The backbone of the 10-residue poly-alanine chain built is shown in yellow. Colours of FluPol$_C$ subunits are as in Figure 1.

Related to Figure 1.

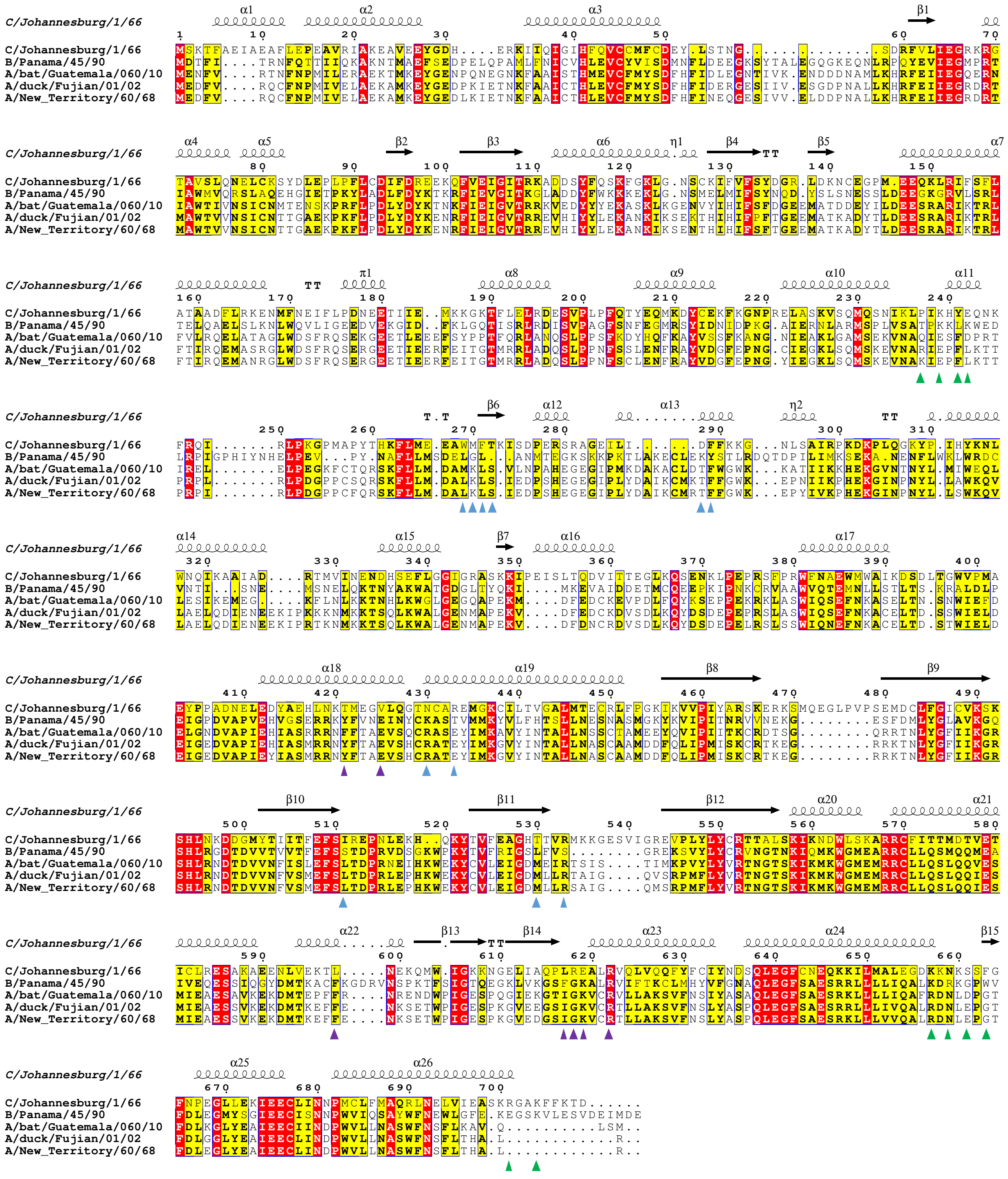

Figure S2. Amino acid sequence alignment of FluPol PA/P3 subunit.

Amino acid sequence alignment for the PA/P3 subunit from influenza C/Johannesburg/1/66, B/Panama/45/90, A/bat/Guatemala/060/10, A/duck/Fujian/01/02 and A/New Territory/60/68. Highly conserved residues are shown in red, partially conserved residues in yellow. Amino acid numbering and secondary structure correspond to C/Johannesburg/1/66. Residues proximal to FluPol$_C$ pS$_5$-CTD binding site 1 are highlighted by green arrows. The residues in FluPol$_A$ and FluPol$_B$ PA involved in pS$_5$-CTD binding reported by Lukarska *et al.*, 2017 are highlighted. Purple arrows indicate residues at site 1, which are conserved in influenza A and B viruses; blue arrows indicate residues at site 2, conserved only in influenza A viruses.

Related to Figure 1 and Figure 2.

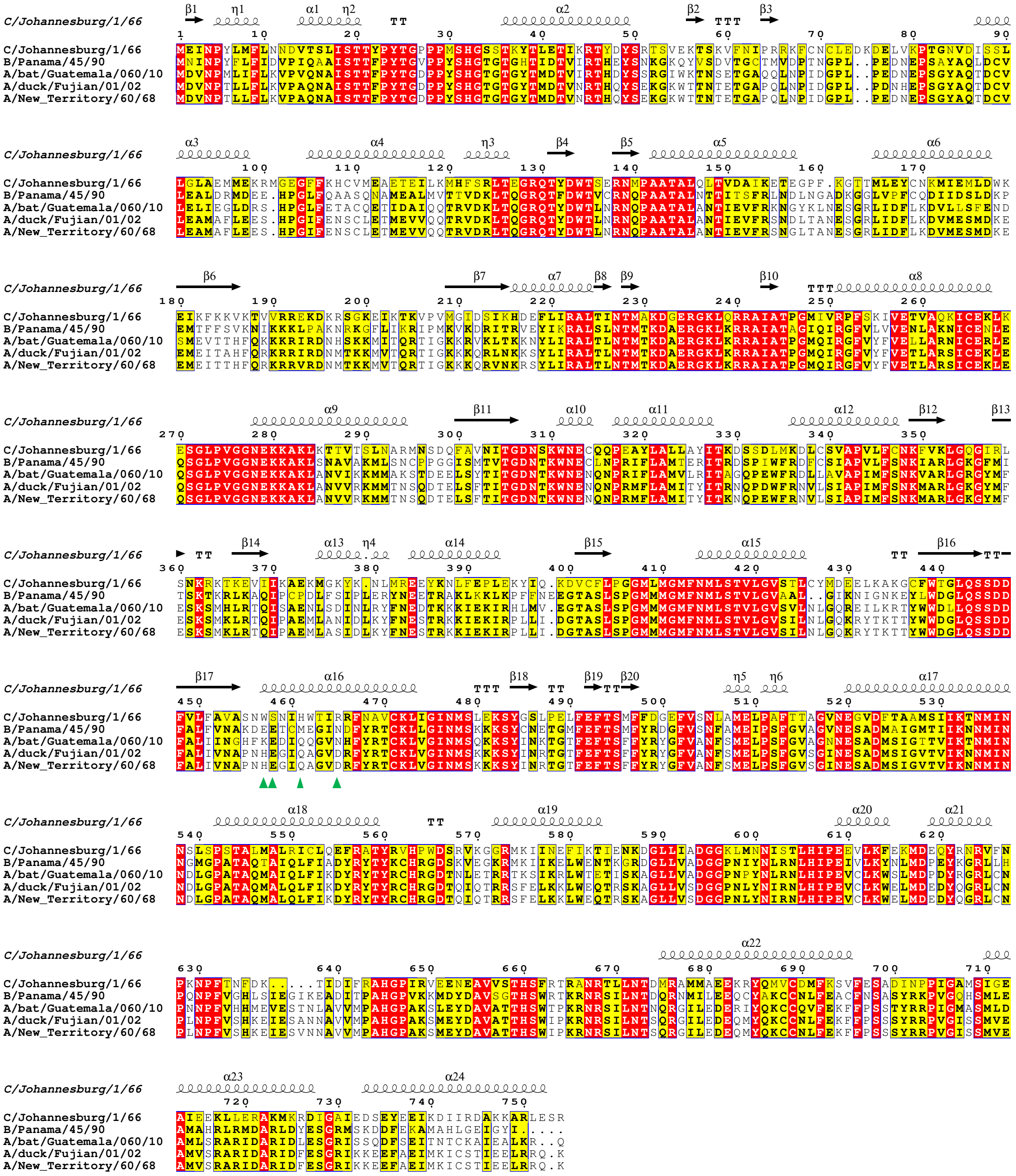

Figure S3. Amino acid sequence alignment of FluPol PB1 subunit.

Amino acid sequence alignment for the PB1 subunit from influenza C/Johannesburg/1/66, B/Panama/45/90, A/bat/Guatemala/060/10, A/duck/Fujian/01/02 and A/New Territory/60/68. Highly conserved residues are shown in red, partially conserved residues in yellow. Amino acid numbering and secondary structure correspond to C/Johannesburg/1/66. Residues proximal to $FluPol_C$ pS_5-CTD binding site 1 are highlighted by green arrows.

Related to Figure 1 and Figure 2.

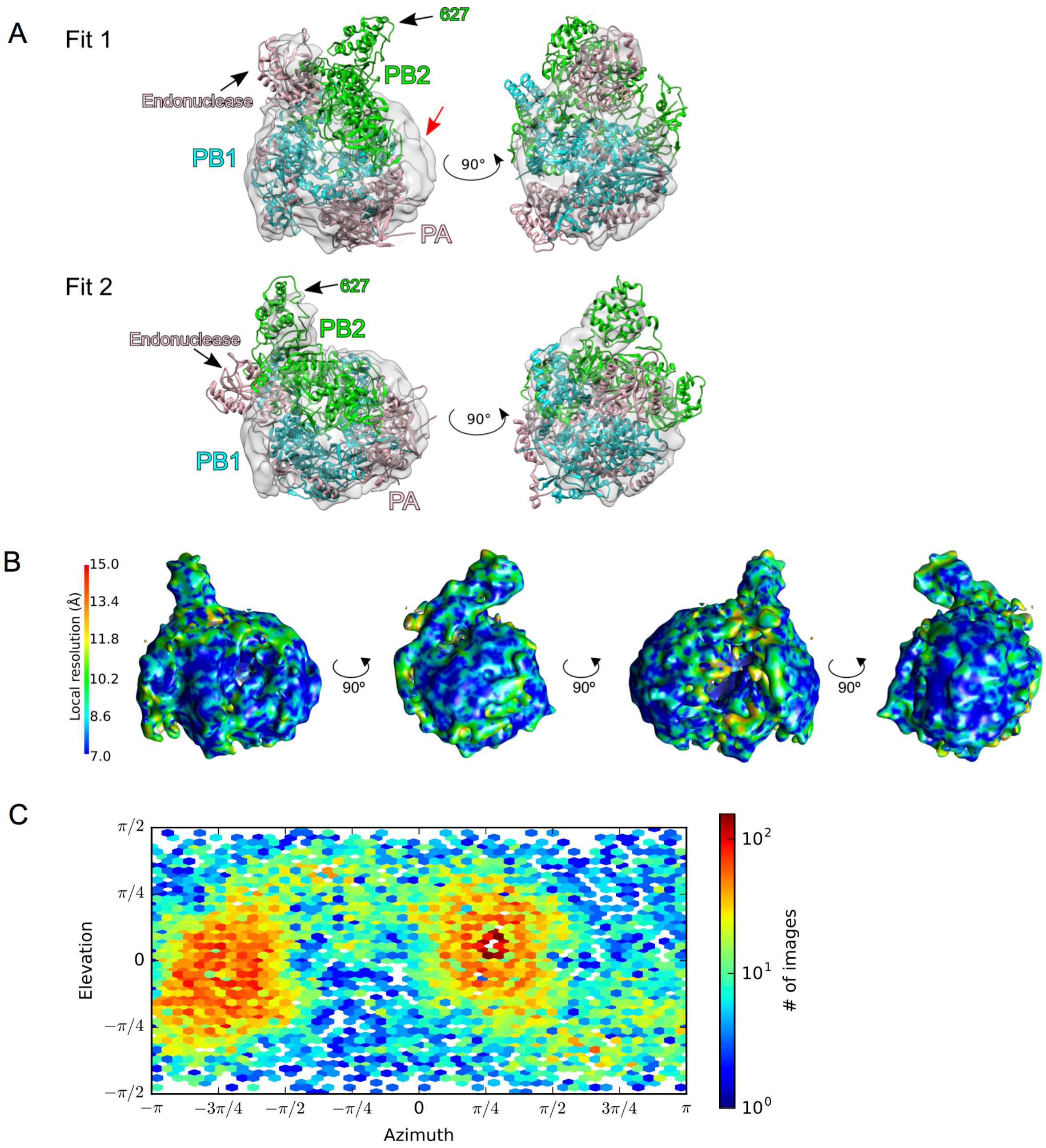

Figure S4. Cryo-EM analysis of FluPol$_C$ bound to vRNA promoter.

(A) The structure of apo FluPol$_C$ in transcriptionally inactive conformation (PDB: 5D98) was fit into the cryo-EM density of FluPol. Two possible fits of the crystal structure are shown: in fit 1, the PA endonuclease domain is placed inside a small peripheral density, which leaves large areas of the density volume unoccupied (red arrow); in fit 2, the PB2 627 domain is placed inside the small density, leaving the endonuclease domain protruding outside the density.

(B) Local resolution of the vRNA promoter bound FluPol$_C$ cryo-EM density map. Note that the third panel shows a distinct patch of low resolution.

(C) Particle orientation distribution in the vRNA promoter bound FluPol$_C$ cryo-EM dataset showing two main populations with reduced coverage of other views. Orientation distribution plot generated in cryoSPARC (Punjani et al., 2017).

Related to Figure 3.

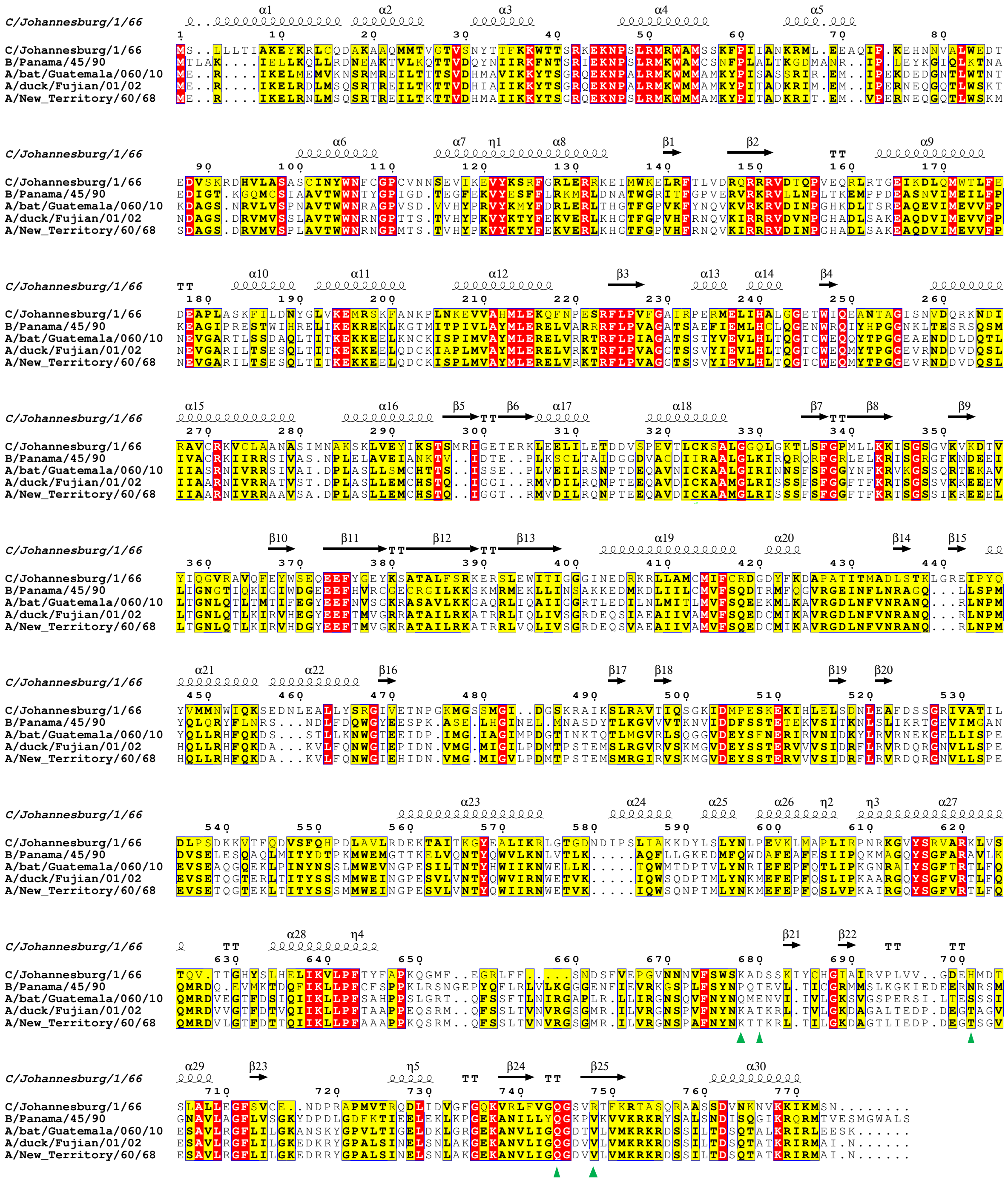

Figure S5. Amino acid sequence alignment of FluPol PB2 subunit.

Amino acid sequence alignment for the PB2 subunit from influenza C/Johannesburg/1/66, B/Panama/45/90, A/bat/Guatemala/060/10, A/duck/Fujian/01/02 and A/New Territory/60/68. Highly conserved residues are shown in red, partially conserved residues in yellow. Amino acid numbering and secondary structure correspond to C/Johannesburg/1/66. Residues proximal to FluPol$_C$ pS$_5$-CTD binding site 1 are highlighted by green arrows.

Related to Figure 4 and Figure 5.

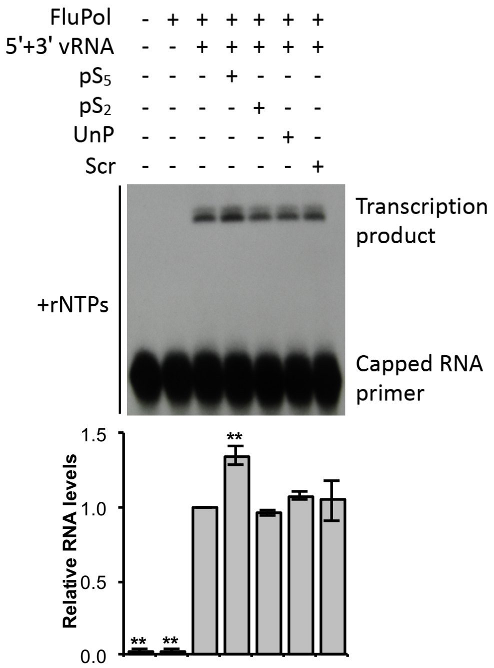

Figure S6. Effect of Pol II CTD peptides on FluPol$_A$ transcriptional activity.

In vitro cap-dependent transcription initiation assay performed using purified FluPol$_A$ in the presence of pS$_5$, pS$_2$, un-phosphorylated (UnP) or scrambled (Scr) Pol II CTD peptides. Assay was carried out in the absence or presence of 5' and 3' vRNA promoter RNAs. The mean of three independent experiments is shown with error bars representing standard deviation. Asterisks indicate a significant difference from the reaction in absence of CTD peptide (**$P < 0.01$, based on a two-sample *t*-test).

Related to Figure 6.
